# Comparison of the Total, Diazotrophic and Ammonia-Oxidizing Bacterial Communities Between Under Organic and Conventional Greenhouse Farming

**DOI:** 10.3389/fmicb.2020.01861

**Published:** 2020-08-12

**Authors:** Chen Chen, Hui Han, Ting Xu, Yizhong Lv, Kelin Hu, Xue Xian Li, Yuhui Qiao, Guo-Chun Ding, Ji Li

**Affiliations:** ^1^Beijing Key Laboratory of Biodiversity and Organic Farming, College of Resources and Environmental Sciences, China Agricultural University, Beijing, China; ^2^Organic Recycling Institute (Suzhou) of China Agricultural University, Suzhou ViCheck Biotechnology Co., Ltd., Suzhou, China

**Keywords:** organic greenhouse farming, bacteria, diazotrophs, ammonia-oxidizing bacteria, high-throughput sequencing

## Abstract

Organic greenhouse farming is an innovative system that may maintain a high yield and healthy agroecosystem. There have been no rigorous studies on the comparison of total and nitrogen-cycling bacterial community in vegetable soils between organic and conventional farming management at large scale. A survey of bacterial community and nitrogen cycles from soils under organic and conventional greenhouse farming was performed at 30 sites, covering seven soil types with 4 to 18 years of organic farming history. Communities of the total, diazotrophs and ammonia-oxidizing bacteria were studied with high-throughput sequencing of the 16S *rRNA*, *nifH* and *amoA* genes, respectively. Organic greenhouse farming did not influence alpha diversities. Beta diversities among the total (26/30) and diazotrophic (17/19) bacteria differed between farming systems, but compositional differences in ammonia-oxidizing bacteria between the two farming systems were only detected at 6 sites. Despite the effects of farming system on most bacterial genera were varied across different sites, organic greenhouse farming persistently selected for a few genera, possibly for the biodegradation of organic carbon with high molecular weight (*Hyphomicrobium, Rubinisphaera, Aciditerrimonas, Planifilum, Phaselicystis*, and *Ohtaekwangia*), but against putative ammonia oxidizing (*Nitrosospira, Nitrosopumilus*) and diazotrophic (*Bradyrhizobium*) bacterial genera, as determined by 16S *rRNA* analysis. Diazotrophic bacteria affiliated with *nifH* cluster 1J were preferentially associated with organic greenhouse farming, in contrast to *Paenibacillus borealis*. In summary, this study provides insights into the complex effects of organic greenhouse farming on the total, diazotrophic and ammonia oxidizing bacterial communities across different environmental context.

## Introduction

In China, greenhouse vegetable production increased rapidly, with a yield of ca. 260 million tons in 2014, accounting for 35% of the total vegetable production (National Bureau of Statistics of the People’s Republic of China [NBSC], 2014; Zhong Jing Hui Cheng Institute of Urban-Rural Planning and Design [ZIURPD], 2014). Intensive agricultural management, such as continuous cropping and overuse of chemical fertilizers, pesticides, irrigation or tillage, can maintain high but short-term productivity. However, soil degradation (Hannachi et al., 2015), severe greenhouse gas emissions (Alam et al., 2019), contamination of groundwater (Yang et al., 2018), loss of organic matter (Scotti et al., 2015), and accumulation of pesticides (Chen et al., 2015) frequently lead to failure of sustainable production, especially under greenhouse conditions. Adaptation of organic agricultural management into greenhouse vegetable production may mitigate these adverse environmental problems. In general, organic greenhouse farming delivered greater ecological services but smaller (between 5 and 34%) productivity than conventional farming (Bengtsson et al., 2005; Seufert et al., 2012). Due to price premiums for organic foods, organic farms could still achieve income levels similar to or higher than those of comparable conventional farms (Qiao et al., 2018). In China, the total area for organic vegetable production reached 148,500 hectares by 2017, accounting for 6% of the commercial vegetable planting area^1^. Although whether organic greenhouse farming can mitigate climate warming is controversial (Searchinger et al., 2018; Smith et al., 2019), the positive effects of organic greenhouse farming on ecosystem functions such as soil fertility (Gomiero et al., 2011) and plant health (Van Bruggen et al., 2016) are very promising.

Soil bacteria, providing the majority of biodiversity in soil ecosystems, participate in almost every crop-soil interaction, including in soil carbon and nitrogen cycling (Thorpe and Callaway, 2011; Che et al., 2018), plant growth and health (Janvier et al., 2007; van Bruggen et al., 2015), and the maintenance of soil architecture (Bonanomi et al., 2016). For example, aerobic ammonia oxidation mediated by β- and γ-proteobacteria is the rate-limiting step in nitrification, which is associated with nitrogen availability to plants, the leaching of nitrate into groundwater or N_2_O emission (Di and Cameron, 2016). Diazotrophic bacteria can transform atmospheric N_2_ into a biologically useful form, and the *nifH* gene encoding a subunit of nitrogenase reductase (Kuypers et al., 2018) is often used as a genetic marker for the molecular analysis of N-fixing bacteria (Levy-Booth et al., 2014). Functional populations involved in nitrogen fixation or nitrification are associated with soil fertility and high nitrogen use efficiency (Levy-Booth et al., 2014). Free-living or symbiotic diazotrophic bacteria contribute to the gaining of biological useful nitrogen while ammonia-oxidizing bacteria (AOB) or ammonia-oxidizing archaea (AOA) could oxidize ammonia into nitrite and further into nitrate which can easily loss during leaching. Here, we studied greenhouse agriculture where excessive nitrogen was often applied to maintain high vegetable productivities, AOB was selected as indicative microbial populations due to it were more responsive to higher N inputs (Hink et al., 2018).

The effects of organic farming on the soil microbiome are complex. Previously, its effects on the taxonomic compositions of the soil microbiome have been explored under field (Dennert et al., 2018) and greenhouse (Hartmann et al., 2015; Ding et al., 2019) conditions. Organic farming enhances soil microbial abundance and activity (Lori et al., 2017; Ouyang et al., 2018b) and shifts microbial community compositions (Moeskops et al., 2010; Bonanomi et al., 2016). However, the effects on microbial alpha diversity (Lupatini et al., 2017; Chen et al., 2018) and specific taxonomic groups (Tuck et al., 2014) has varied between studies (Postma et al., 2008; Bonanomi et al., 2016) and across time (Wu et al., 2019). The effects of organic fertilizer on the diversity and abundance of ammonia-oxidizing bacteria have been evaluated extensively (Chu et al., 2007; Ouyang et al., 2018a). The abundance or relative abundance of ammonia oxidizing bacteria tends to be lower in organic than conventional or integrated farming systems (Wessen et al., 2011; Ding et al., 2019). In a farm-scale field study, the community composition of ammonia oxidizing bacteria and archaea showed no difference between organic and integrated farming systems (Wessen et al., 2011). In a greenhouse experiment, organic farming altered the ammonia-oxidizing bacterial community (Ding et al., 2019). Agricultural management may enrich the abundance and diversity of diazotrophic bacteria (Köberl et al., 2016), but a direct comparison between organic and conventional farming systems is rare (Ding et al., 2019). The effects of organic greenhouse farming on soil microbial diversity are likely to be context dependent. Here, we hypothesized that organic greenhouse farming may still cause persistent, indicative changes in total, diazotrophic and ammonia oxidizing bacterial communities in the soil microbiome across different environmental context. This knowledge is significant to deepen our understanding of the mechanisms associated with ecological services delivered by organic greenhouse farming.

In the present study, a survey including thirty sites in China was performed in July 2017 to study the interplay between organic greenhouse farming and the soil microbiome. The diversities of total, diazotrophic and ammonia-oxidizing bacteria were explored by high-throughput sequencing analyses of PCR amplified 16S *rRNA, nifH* and *amoA* gene fragments. The aims of the present study were to (1) examine the interplay between organic greenhouse farming and total, diazotrophs, and ammonia-oxidizing bacteria across different sites; (2) identify the key factor underlying the interplay; and (3) identify persistent, predictable changes in those microbial populations.

## Materials and Methods

### Soil Sampling

**FIGURE 1:**
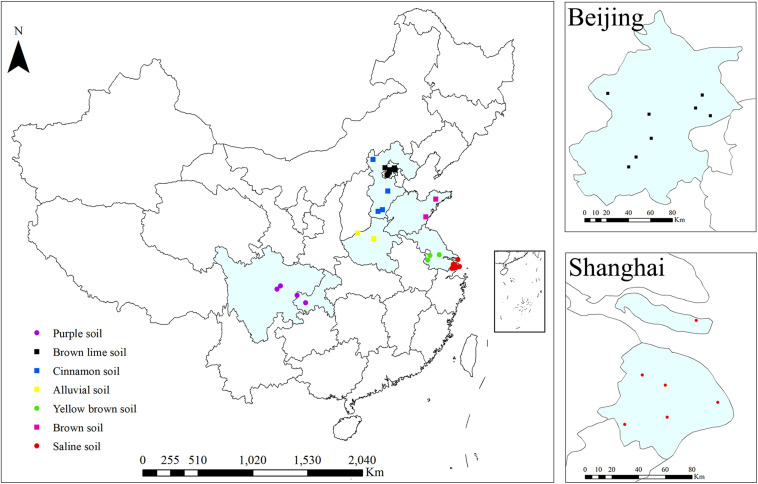
Locations of organic and conventional farms for greenhouse vegetable production. The colors on the points indicate soil types: black (Beijing, brown lime soil), blue (Hebei, cinnamon soil), yellow (Henan, alluvial soil), magenta (Shandong, brown soil), green (Jiangsu, yellow brown soil), red (Shanghai, saline soil) and purple (Chengdu/Chongqing, purple soil).

A total of 30 sites located in the regions with the most organic farms in China were selected (Figure 1). These sites represent 4 to 18 years of organic greenhouse management and seven soil types. Eight and six sites were in Beijing and Shanghai, respectively (Figure 1). All studied organic farms were certified. Organic greenhouse farming was performed according to the basic standards of the International Federation of Organic Agriculture Movements (IFOAM) by using biological and physical methods for plant protection and organic fertilizers (farmyard manure and commercial organic fertilizer) (Table 1). Frequently, both organic and mineral fertilizers were used in conventional farms, in which chemical agents for weed (glyphosate and oxalamine), disease (azoxystrobin and thiophanate methyl) and insect (chlorfenapyr and lufenuron) control as well as synthetic plant growth regulators were applied (Table 1). For each site, three adjacent conventional and organic greenhouses with the same crop (tomato, cucumber, eggplant, or green pepper), the same soil types and similar cropping rotations, were selected by interviewing local farmers. For each replicate, ten drills of topsoil (0–20 cm) were collected (approximately 1 kg). Samples were transported to the lab in a cool box and kept at −20°C prior to DNA extraction, which was performed after sieving the samples through a 2-mm mesh. The soil physicochemical properties total nitrogen (TN), total carbon (TC), pH, electrical conductivity (EC), density, available nitrogen (AN), Olsen phosphorous (Olsen P), available potassium (K) and cation exchange capacity (CEC) were determined according to standard protocols (Bao, 2000).

**TABLE 1:** Details of agricultural managements under the long-term greenhouse experiments.

**Farming systems**	**Organic**	**Conventional**
Vegetable species	Leafy vegetables (green vegetables and leek), Melons and fruits (tomatoes, cucumbers, eggplants, and green peppers)	Leafy vegetables (green vegetables and leek), Melons and fruits (tomatoes, cucumbers, eggplants, and green peppers)
Fertilization types	Seedling medium, commercial organic fertilizer, liquid fertilizer, natural mineral fertilizer and composting fertilizer	Compost, urea, calcium superphosphate and potassium chloride, compound fertilizer
**Plant protection**		
Insects	Sticky yellow paper traps and insect net, natural botanical pesticide (pyrethrin, matrine)	Insect net, boscalid, acetamiprid, prochloraz manganese salt, chlorfenapyr
Plant diseases	Biopesticide, mechanical removed diseased plants, pest and disease resistant varieties, ridging planting	Biocontrol, bactericide, ridging planting
Weed	Artificial/mechanical weed removing	Artificial/mechanical weed removing, herbicide
Measures to improve soil fertility	Returning straw to field, green manure, covering, high temperature stuffy shed, plowing	Returning straw to field, green manure, covering, high temperature stuffy shed, plowing
Irrigation methods	Flood irrigation, spray irrigation, trickle irrigation	Flood irrigation

### PCR Amplification of 16S *rRNA, amoA*, and *nifH* Genes

Total community DNA was extracted using the FastDNA Spin Kit for Soil (MP Biomedicals, Santa Ana, Carlsbad, CA, United States) according to the manufacturer’s instructions. The primers used for amplification of the 16S *rRNA*, *amoA* and *nifH* genes are given in Supplementary Table S1 (Rotthauwe et al., 1997; Poly et al., 2001; Tamaki et al., 2011). PCR products were gel purified, quantified and pooled, with equimolar amounts for each sample, for high-throughput sequencing on the Illumina Hi-Seq platform by using the Reagent Kit v2 2 × 250 bp (Hi-Seq platform 2500).

### Bioinformatic and Statistical Analyses

Sequences of high quality (length >300 bp, without ambiguous base “N,” and average base quality score >30) were used for downstream analyses. Sequences of the 16S *rRNA* and *nifH* genes were assembled using the software package mothur v1.39 (Schloss et al., 2009) and were further assigned to each sample based on barcodes and primers. Bidirectional sequences of the *amoA* genes were assigned to samples separately due to insufficient read length. Denoising, OTU assignment, and classification of the 16S *rRNA* gene were performed as previously described (Schloss et al., 2009; Caporaso et al., 2010; Cole et al., 2013; Ding et al., 2012, 2019). For the *nifH* and *amoA* genes, a standalone BLASTX analysis was performed against corresponding functional gene sequences downloaded from the RDP database to identify correct translation frames. Only those sequences with no stop codon within their deduced amino acid sequences were included for further analysis. Deduced amino acid sequences were further subjected to hmmscan analysis to identify *nifH* or *amoA* gene sequences. The hmm profile was acquired from the RDP FunGene website^2^. Amino acid sequences of *nifH* were further assigned to OTUs (>95% sequence identity) using vsearch software. Representative OTUs of *nifH* were assigned to different subgroups using the curated database as described by Zehr et al. (2017). For *amoA* genes, the selected sequence was grouped into OTUs at 80% identity by analyzing representative sequences of different subgroups as described previously (Li et al., 2011; Guo et al., 2017). Discriminative taxa, OTUs and differences in community composition and co-occurrence network were mainly examined as described previously (Hothorn et al., 2008; Li et al., 2019). Briefly, significant differences in microbial community composition were compared by permutation test (Kropf et al., 2004) using the calculated pairwise Bray-Curtis distance. Based on the “Vegan” package, alpha diversity indexes (Chao1, Simpson, and Pielou’s evenness) were calculated by 1000 re-samplings of an equal amount of sequences from each sample to alleviate biases caused by read number or individual sampling. Comparisons of community composition, identification of taxa with significantly different relative abundances and network analysis were performed as previously described (Li et al., 2019). Classification random forest analyses were also performed with the R-addon package “randomForest” (Liaw and Wiener, 2002) to identify key taxa for farming systems. This network was analyzed using Gephi (version 0.91) software (Bastian et al., 2009). All statistical analyses and plotting were performed with R 3.1.2 software^3^, and these tools were implemented in a Galaxy instance^4^.

## Results

### Effects of Organic Greenhouse Farming on the Alpha and Beta Diversities of Soil Bacteria

**FIGURE 2:**
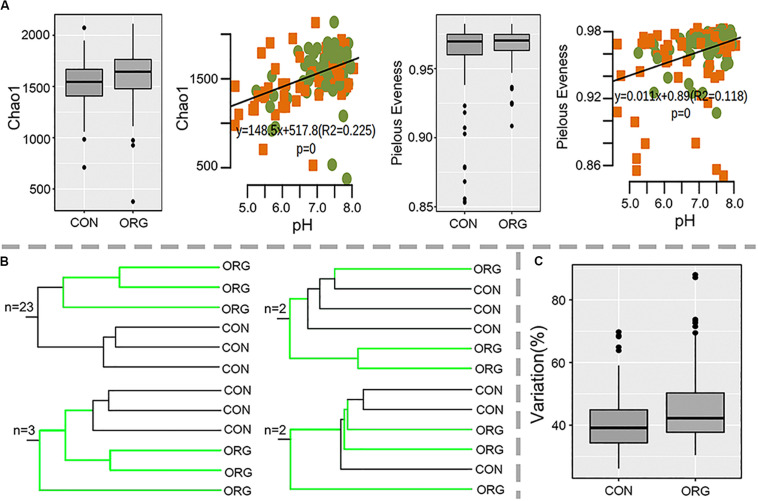
Alpha- and beta-diversity of bacterial microbial communities in soils under organic (ORG) and conventional (CON) farming systems at 30 sites. **(A)** Chao1 and Pielou’s evenness indexes and their correlations with pH; green dots and orange squares indicate organic and conventional farming, respectively. **(B)** Number of sites with the schematic UPGMA cluster. **(C)** Variation in beta diversity within ORG or CON farming systems.

Soil bacterial diversity from organic and conventional farms at 30 sites was studied by 16S *rRNA* sequencing. Proteobacteria, Acidobacteria, Actinobacteria, Firmicutes, Bacteroidetes, Chloroflexi, Planctomycetes, Gemmatimonadetes, and Cyanobacteria were dominant in all the soil samples (Supplementary Figure S1). Alpha diversity (Chao1 richness and Pielou’s evenness index) was not affected by organic greenhouse farming and was significantly correlated with soil pH (Chao1: *R*^2^ = 0.23, *P* = 0; Pielou’s evenness: *R*^2^ = 0.12, *P* = 0) (Figure 2A). Beta diversity clearly differed between organic and conventional farming systems at 26 out of the 30 studied sites (Figure 2B and Supplementary Figure S2). Permutation analysis also confirmed that the bacterial community was significantly (*p* = 0; *d* = 20.2%) different between organic and conventional farming systems. However, the average dissimilarity between organic and conventional farming systems did not increase with the period of organic farming (Supplementary Figure S3). The heterogeneity in community composition was slightly higher in organic than conventional farming system (Figure 2C).

### Common and Site-Specific Taxa Associated With Organic Greenhouse Farming

**FIGURE 3:**
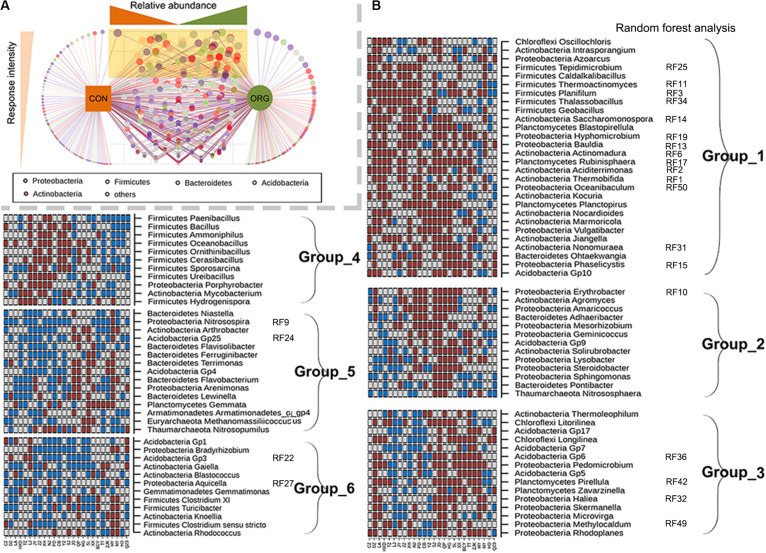
Bacterial genera with significantly (*p* < 0.05) different relative abundances between farming systems and their association with different soil physicochemical properties. **(A)** The frequency of a genus to be discriminative between farming systems. The light yellow triangle indicates an increased frequency of a genus to be discriminative between farming systems. **(B)** Groups of genera with similar response patterns to farming systems and which ranked in the 50 most influential genera across sites; ORG, organic farming system; CON, conventional farming system. Brown and cyan squares indicate significantly (*p* < 0.05) higher or lower relative abundance in the ORG than in the CON, respectively. Gray squares indicate no significant difference between farming systems.

Genera with significantly (*P* < 0.05) different relative abundances between farming systems were identified for those 26 sites with different community compositions between farming systems. The effects of organic greenhouse farming on most genera were inconsistent, as demonstrated by the low frequency or site-dependent response to farming systems (Figure 3A). Ninety-four genera that frequently (>12 sites) differed in relative abundance between farming systems were further analyzed for their response patterns (Figure 3B). Despite the wide geographical distribution of the studied sites, genera that were preferentially (Group 1) or negatively (Group 5 and 6) associated with organic greenhouse farming were still identified (Figure 3B). *Hyphomicrobium, Rubinisphaera, Aciditerrimonas, Planifilum, Phaselicystis*, and *Ohtaekwangia*, known as chemoorganotrophic microorganisms, were preferentially associated with organic greenhouse farming (Figure 3B). *Bradyrhizobium, Nitrosospira, Nitrosopumilus, Gaiella, Arthrobacter, Flavisolibacter, Acidobacter Gp1, GP3, Gp4*, and *Gp25* were negatively associated with organic greenhouse farming (Figure 3B). Interestingly, 23 of these genera ranked as the top 50 genera that were the most influential between farming systems as revealed by random forest analysis (Figure 3B). Notably, seventeen of them demonstrated persistent response patterns to organic farming (Figure 3B). These results suggested that organic greenhouse farming tended to select for organic matter-degrading bacteria but against diazotrophic, ammonia-oxidizing, and oligotrophic bacteria.

### Diversity and Community Composition of Diazotrophic Populations

**FIGURE 4:**
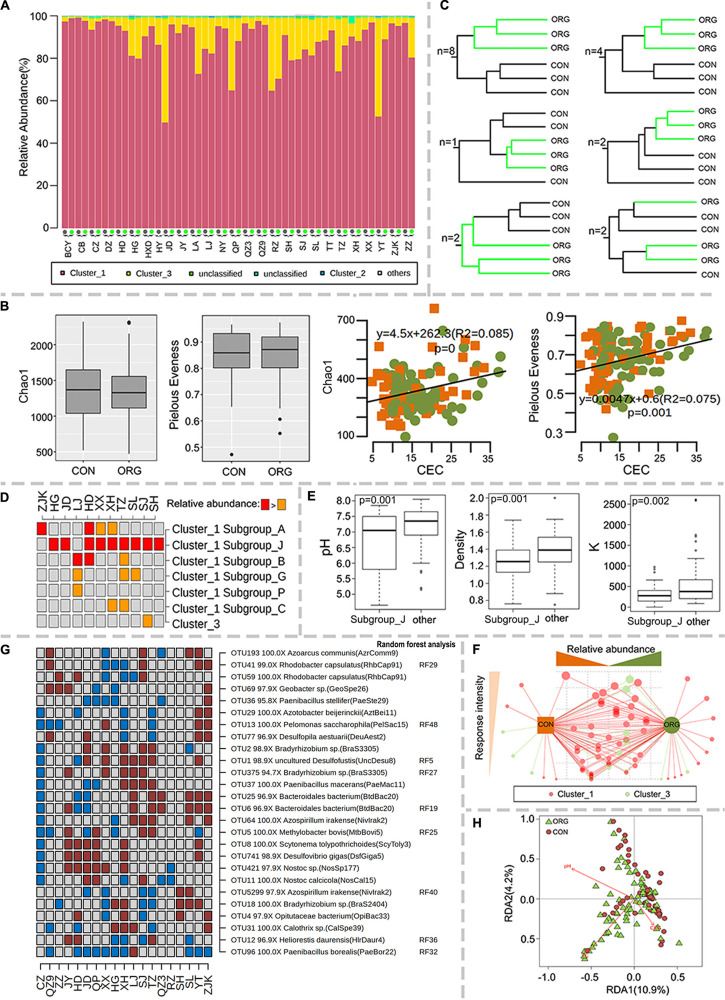
Association of the diversity of diazotrophic populations with the farming system. **(A)** Relative abundance of major *nifH* gene clusters, **(B)** Chao1 and Pielou’s evenness indexes and their correlations with CEC; green dots and orange squares indicate organic and conventional farming, respectively. **(C)** Number of sites with the schematic UPGMA cluster. **(D)** Discriminative subgroups of *nifH* genes. **(E)** Boxplot of differences in pH, density and K levels between the sites with increased and non-increased relative abundance of subgroup J of *nifH* cluster 1. **(F)** The association of each diazotrophic OTU with the farming system. The size of the dots indicates the frequency of each OTU to be discriminative between farming systems. The tendency of each OTU to organic or conventional farming systems was indicated by their distance to farming systems. **(G)** Discriminative diazotrophic OTUs between farming systems ranked in the 50 most influential OTUs. Brown and cyan squares indicate significantly (*p* < 0.05) higher or lower relative abundance in the ORG than in the CON, respectively. Gray squares indicate no significant difference between farming systems. **(H)** Redundancy analysis (RDA) of soil characteristics and diazotrophic microbial communities. ORG, organic farming system; CON, conventional farming system.

The diversity of diazotrophic bacteria was studied by high-throughput sequencing analysis of the *nifH* gene. The effects of organic greenhouse farming were only successfully studied at 19 sites where sufficient sequences were acquired for at least two samples per farming system. Most sequences fell within *nifH* clusters 1 and 3 (Figure 4A), which were mainly affiliated with Proteobacteria, Cyanobacteria and Firmicutes. Alpha diversity (Chao1 richness and Pielou’s evenness indexes) was not affected by organic greenhouse farming (Figure 4B) but was significantly correlated with CEC (Chao1: *R*^2^ = 0.09, *P* = 0; Pielou’s evenness: *R*^2^ = 0.08, *P* = 0.001) (Figure 4B). The community composition of diazotrophic populations differed between organic and conventional farming at 17 out of 19 sites (Figure 4C and Supplementary Figure S4). The relative abundance of subgroup J of *nifH* cluster 1 was significantly high in the soil organically managed at nine sites (Figure 4D), where soil pH, K and density were significantly lower than those at other sites (Figure 4E). The relative abundances of cluster 3 and subgroup G and C of cluster 1 tend to be negatively associated with organic greenhouse farming (Figure 4D). A divergent association of subgroup A of cluster 1 with organic greenhouse farming was observed (Figure 4D). The most dominant (top 5) OTUs in each sample were selected to identify key diazotrophic populations that responded to organic greenhouse farming. Seventy out of 81 OTUs varied between the two farming systems at one or more sites, and a majority of these OTUs were identified as *nifH* cluster 1 or 3 (Figure 4F). Interestingly, the relative abundance of the *nifH* gene affiliated with *Paenibacillus borealis* was negatively associated with organic greenhouse farming (Figure 4G). Several other diazotrophic populations were divergently associated with organic greenhouse farming (Figure 4G). The putative lifestyles of these populations are diverse, including symbiotic (*Bradyrhizobium*), free-living (*Azoarcus, Paenibacillus*, and *Bacteroidales*) and anaerobic (*Desulfofustis, Desulfovibrio*) lifestyles (Figure 4G). Redundancy analysis (RDA) indicated that soil pH, TC, and CEC could explain most of the variations in those OTUs on a large scale (Figure 4H).

### Diversity and Community Composition of Ammonia-Oxidizing Populations

**FIGURE 5:**
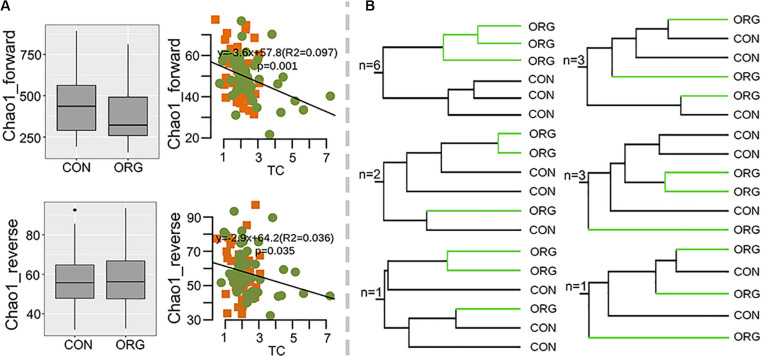
Association of the diversity of ammonia-oxidizing bacteria with the farming system. **(A)** Chao1 indexes of ammonia-oxidizing bacteria as revealed by forward and reverse datasets and their correlations with TC; green dots and orange squares indicate organic and conventional farming, respectively. **(B)** Number of sites with the schematic UPGMA cluster. Brown and cyan squares indicate significantly (*p* < 0.05) higher or lower relative abundance in the ORG than in the CON, respectively. Gray squares indicate no significant difference between farming systems. ORG, organic farming system; CON, conventional farming system.

Here, the effects of organic greenhouse farming on ammonia-oxidizing bacteria were analyzed at 16 sites. The diversity of *amoA* gene sequences was also high, reaching 6,769 OTUs (>80% identity), with the majority affiliated with *Nitrosospira, Nitrosovibrio and Nitrosomonas-*like bacteria. Again, the alpha diversity of *amoA* was not influenced by organic greenhouse farming, and Chao1 was significantly correlated with TC (forward: *R*^2^ = 0.097 *P* = 0.001; reverse: *R*^2^ = 0.036, *P* = 0.035) (Figure 5A). The community composition of ammonia oxidizing bacteria only differed between organic and conventional farming systems at six sites (Figure 5B and Supplementary Figures S5, S6). *AmoA* OTUs affiliated with *Nitrosovibrio-*like bacteria tended to be negatively associated with organic greenhouse farming, in contrast to those *Nitrosospira*-like bacteria (Supplementary Figure S7). These results indicated that the effects of organic greenhouse farming on the composition of ammonia oxidizing bacteria were patchy.

### Associations Among Total, Diazotrophic and Ammonia-Oxidizing Populations and Test-Site Physicochemical Properties

**FIGURE 6:**
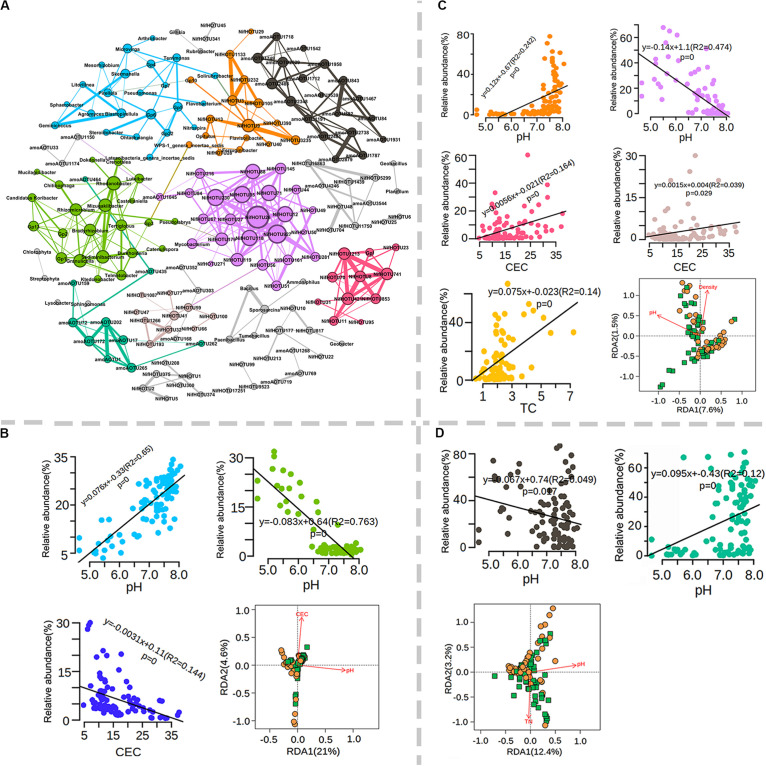
Co-occurrence network analysis of dominant total, diazotrophs and ammonia oxidizing bacteria. **(A)** Co-occurrence network and major microbial hubs as indicated by different colors. Number and fraction of cooccurring microbial populations; microbial hubs association with soil physicochemical properties for total **(B)**, diazotrophs **(C)** and ammonia-oxidizing bacteria **(D)**.

It is known that soil microbial communities are sensitive to environmental changes such as physicochemical characteristics and climate change and result in differences in microbial communities of test sites. Dominant (>1% relative abundance) total (112), diazotrophic (177) and ammonia-oxidizing (97) bacterial OTUs were subjected to co-occurrence network analysis (Supplementary Figure S8). On average, 37.3% of the selected taxa exhibited significant co-occurrence with each other, and these genera were mainly affiliated with Proteobacteria (16), Acidobacteria (13), acteroidetes (6), Firmicutes (5), Actinobacteria (4) and Chloroflexi (3) (Supplementary Figure S8). Among these phyla, the highest fraction of co-occurring genera was affiliated with Acidobacteria, a phylum frequently referred to as k-strategists (Supplementary Figure S8). These co-occurring microbial populations formed 11 major microbial hubs (more than five nodes) by network analysis (Figure 6A). Interestingly, total taxa and diazotrophic and ammonia-oxidizing bacteria mainly formed separate hubs, suggesting that the dominant bacteria exhibited low correlation with both functional populations (Figure 6A). The relative abundance of different hubs varied greatly among sites but exhibited little association with farming systems (Supplementary Figures S9A–C). Soil pH and CEC were two main factors affecting the relative abundances of three major total bacterial hubs (green, cyan, and blue) (Figure 6B). Soil pH, CEC, TC, TN, and EC values were closely associated with the composition of diazotrophic bacteria, among which five major hubs were significantly correlated with pH, TC or CEC (Figure 6C). Interestingly, the relative abundance of the orange hub tended to be high under neutral pH conditions, in contrast to that of the purple hub (Figure 6C). In addition to pH, two hubs (red or brown) were positively correlated with CEC, and the yellow hub was positively correlated with TC (Figure 6C). Dominant OTUs of ammonia-oxidizing bacteria formed two major hubs (Figure 6D). The relative abundance of the green hub tended to decrease when the pH was lower than 6.5 (Figure 6D), while the opposite trend was observed for the black hub (Figure 6D). RDA further revealed that soil pH explained the significant variation within co-occurring populations for all three populations (Figures 6B–D). These results indicate that soil pH was a key factor shaping microbial occurrence in the total, diazotrophic and ammonia-oxidizing bacterial communities.

## Discussion

Understanding the interplay between organic farming and the soil microbiome is important for enhancing the ecological benefits of organic farming for the sustainable production of food. Here, a large survey across soils with a broad range of physicochemical properties, geographical regions and organic farming periods was performed to examine the effects on total, diazotrophic and ammonia oxidizing bacterial diversity, which provides an opportunity to examine the effects of organic farming on the soil microbiome. In addition, organic greenhouse farming may cause persistent, predictable changes in total, diazotrophic and ammonia oxidizing bacterial communities across different soils. This knowledge is significant to deepen our understanding of organic farming on the soil microbiome under greenhouse conditions across space.

### Organic Farming Shifted Bacterial Community Composition, but It Did Not Affect Alpha Diversity or Increased Community Heterogenicity Under Greenhouse Condition

Previously, the effects of organic farming on soil microbial communities have been detected under both greenhouse and field conditions for a few studied sites (Hartmann et al., 2015; Bonanomi et al., 2016; Ding et al., 2019). However, spatial and temporal variation are common for soil microbial communities (Uksa et al., 2014; Hill et al., 2016); thus, it is still uncertain whether organic greenhouse farming can cause changes in soil microbial diversity. Here, distinct bacterial compositions between organic and conventional greenhouse farming systems were observed for 26 out of 30 sites. These findings indicated that organic greenhouse farming was largely a momentous driver of bacterial communities across different sites. The alpha diversity of soil bacteria did not differ by farming system, in agreement with other studies (Prober et al., 2015; Banerjee et al., 2019). It was significantly correlated with soil pH. The decisive role of pH in the alpha diversity of soil bacteria has been demonstrated previously (Rousk et al., 2010; Wang et al., 2017). Our results showed that the effect of pH on bacterial alpha diversity could also be extended to greenhouse conditions. The heterogenicity in community composition was comparable between farming systems. Other studies suggested that the soil microbiome was more heterogeneous in organic than in conventional farming systems (Tuck et al., 2014; Lupatini et al., 2017). Compared to the field or grassland, agricultural management under greenhouse conditions is highly intensive, and these management practices may reduce heterogenicity in soil.

### Organic Greenhouse Farming Caused Persistent Changes in a Few Bacterial Taxa Across Different Sites

The effects of organic greenhouse farming on most bacterial genera (including groups 2, 3, and 4 in Figure 2B) varied across different sites, as several other factors, such as soil physicochemical properties, types and amount of organic fertilizer, may also affect soil microbial communities (Chu et al., 2007; Yao et al., 2016; Ouyang et al., 2018a). However, we still detected genera that were persistently responsive to organic greenhouse farming. Several genera (group 1 in Figure 3) preferentially associated with organic greenhouse farming were often prevalent in environments with abundant organic materials (Dennert et al., 2018; Zhang et al., 2019). In contrast, other genera, such as *Nitrosospira* and *Bradyrhizobium*, were negatively associated with organic greenhouse farming. Members of *Nitrosospira* were able to perform ammonia oxidization, and changes in putative ammonia-oxidizing bacteria agree with other studies on organic farming (Wessen et al., 2011; Ding et al., 2019). Members of *Bradyrhizobium* were able to form symbioses with legume plants for biological nitrogen fixation (Liu et al., 2015). Furthermore, groups of genera with similar response patterns to farming systems and which ranked in the 50 most influential genera across sites by random forest analysis provide insight into the response of important taxa to organic greenhouse farming.

### Organic Greenhouse Farming Shifted Diazotrophic Bacterial Communities, and Its Effects on Different Diazotrophic Populations Were Largely Site-Dependent

Free-living or symbiotic diazotrophic bacteria can fix atmospheric N_2_ and add biologically active N to agroecosystems (Orr et al., 2011). The alpha diversity of diazotrophic bacteria did not differ by farming system but was correlated with the CEC. In a recent study by Wang (Wang et al., 2019), the highest diversity of diazotrophic bacteria was detected in the soil with the highest CEC. The community composition of diazotrophic bacteria has implications for the rate of nitrogen fixation in agricultural systems (Hsu and Buckley, 2009; Feng et al., 2018). Here, the diazotrophic community differed by farming system at 17 out of 19 sites (The significant difference on the community composition between ORG and CON was confirmed by 1000 times two-way permutation analysis with a *p*-value of 0.000 and the community dissimilarity of 21.8%). Although direct comparisons of diazotrophic communities between organic and other farming systems are rare (Orr et al., 2011; Ding et al., 2019), agricultural management associated with organic farming, such as organic fertilization, has been suggested to be a factor driving diazotrophic bacterial populations including alpha- and beta- diversity (Tang et al., 2017; Wang et al., 2017; Lin et al., 2018).

*NifH* cluster 1 and 3 were dominant in greenhouse soils regardless of the farming system, and their prevalence in terrestrial ecosystems was reported previously (Zehr et al., 2017). The effects of organic farming on different groups of diazotrophs are largely unknown (Grossman et al., 2005; Ramos et al., 2011). Here, we found that *nifH* cluster 1J, frequently harbored by Proteobacteria and Cyanobacteria (Krausfeldt et al., 2017; Zehr et al., 2017), was preferentially associated with organic farming at sites where soil pH, K and density were significantly low. Diazotrophic *P. borealis* was negatively associated with organic farming. Changes in *P. borealis* seem to be inconsistent with the results of 16S *rRNA* gene analysis, in which the association of *Paenibacillus* with organic farming was divergent. It is possible that only a fraction of *Paenibacillus* carries the *nifH* gene (Grube et al., 2009). The effects of organic greenhouse farming on several other diazotrophic bacteria, such as *Bradyrhizobium*, *Azoarcus*, *Paenibacillus*, *Bacteroidales*, *Desulfofustis*, and *Desulfovibrio*, with putative symbiotic, free-living and anaerobic lifestyles were inconsistent across different sites (Wakelin et al., 2010). RDA further revealed that soil pH could explain most of the variation, in agreement with other studies (Reardon et al., 2014; Zarraonaindia et al., 2015; Wang et al., 2017). Organic greenhouse farming possibly exerts selection pressure on diazotrophic bacteria adapted to different niches by altering key soil physicochemical properties.

### The Effects of Organic Greenhouse Farming on Ammonia-Oxidizing Bacteria Were Largely Patchy

Previously, the effects of organic greenhouse farming on the alpha diversity of ammonia-oxidizing microorganisms have only been explored in few studies (Wessen et al., 2011; Ding et al., 2019) and were largely unclear. Here, we found that the alpha diversity of ammonia oxidizing bacteria was not different by farming systems but was negatively correlated with the contents of organic matter. Ammonia oxidizing bacteria are largely lithoautotrophic, and ammonia application is likely to be the most important driver of its community composition (Li et al., 2018). Nitrogen fertilizer-dependent changes in the abundance, diversity and activity of these bacteria were frequently detected in several agroecosystems (Shen et al., 2012; Guo et al., 2017). Responses of AOB to ammonia addition were observed at both microcosm and filed experiments (Daebeler et al., 2015; Ying et al., 2017), highlighting that ammonia addition which is common under conventional farming system might play roles on shifting the AOB community. However, compared with the total and diazotrophic bacterial communities, the effects of organic greenhouse farming on ammonia oxidizing bacteria were unexpectedly inconsistent, as community composition was only different by farming systems at six out of seventeen sites. These results indicated that organic greenhouse farming was not necessarily the major driver of ammonia-oxidizing bacteria. In a farm-scale field study, the community composition displayed a patchy pattern (Wessen et al., 2011). Several other factors, such as EC and density, which were less associated with organic farming may synergistically, shape the communities of ammonia-oxidizing bacteria.

### The Soil pH at the Test Site Was Correlated With the Microbial Hubs of Total, Diazotrophic and Ammonia-Oxidizing Bacteria

Organic farming frequently results in increased diversity of macroorganisms (Wanjiku Kamau et al., 2019); however, the effect of organic greenhouse farming on microbial diversity varies among different studies. In general, soil physicochemical properties such as pH, soil type rather than farming systems were the major factors shaping the alpha diversity of total, diazotrophic and ammonia-oxidizing bacteria in soil on a large scale by variation partition analysis (Supplementary Figure S10). Co-occurrence network analysis revealed that dominant total, diazotrophic and ammonia-oxidizing bacteria mainly formed separate hubs, suggesting that diazotrophic or ammonia-oxidizing bacteria were not closely associated with the dominant total bacteria. Previous studies suggested that the abundances of both ammonia-oxidizing and diazotrophic bacteria were frequently low in bulk soil, as the amount of energy harvested by oxidation of ammonia is very low, and N fixation is highly energy intensive (González-Cabaleiro et al., 2019; Luo et al., 2019). The association between the soil microbiome and the physicochemical properties of soil has been explored largely based on taxonomy (Hansel et al., 2008; Rousk et al., 2010; Zhao et al., 2019), and few persistent correlations were detected. It is possible that there was divergence between the taxonomy and physiological properties of the soil microbiome or that several taxa responded similarly to certain changes. Here, we found that the relative abundance of different microbial hubs was often associated with soil pH, CEC, and TC to a large extent. These findings further suggest that co-occurrence network analysis may also help identify the association of microbial populations as hubs with key soil physicochemical properties or highlight that organic farming may alter microbial interactions by changing soil physicochemical properties.

## Conclusion

In summary, the alpha diversity of total, diazotrophic and ammonia oxidizing bacterial communities did not differ by farming system. The beta diversity of the total and diazotrophic, but not the ammonia oxidizing bacterial communities largely varied between farming systems across different sites. Organic greenhouse farming persistently selected for organic carbon degrading bacteria (*Hyphomicrobium, Rubinisphaera, Aciditerrimonas, Planifilum, Phaselicystis*, and *Ohtaekwangia*) and cluster 1J diazotrophs but not for ammonia oxidizing (*Nitrosospira*, *Nitrosopumilus*) and putative symbiotic diazotrophs (*Bradyrhizobium*), possibly by altering key soil physicochemical properties, such as pH or CEC. These results highlight that organic greenhouse farming manipulates the reassembling of soil microbiome associated with the cycling of soil carbon, biological nitrogen fixation and ammonia oxidation, which plays a key role on maintaining soil fertility.

## Data Availability Statement

The datasets generated for this study can be found in the NCBI with accession numbers PRJNA556799 and PRJNA555468.

## Author Contributions

JL, G-CD, TX, and CC designed the experiments, wrote the manuscript, and analyzed the data. CC and HH performed the experiments. All authors participated in the survey, sample collection of organic greenhouse agriculture in different regions, and reviewed the manuscript.

## Conflict of Interest

TX, G-CD, and JL were employed by Suzhou ViCheck Biotechnology Co., Ltd.

The remaining authors declare that the research was conducted in the absence of any commercial or financial relationships that could be construed as a potential conflict of interest.

## Footnotes

1 http://www.cir.cn/

2 http://fungene.cme.msu.edu/

3 http://www.r-project.org/

4 www.freebioinfo.org

## Supplementary Material

FIGURE S1 Relative abundances of dominant bacteria between farming systems. ORG, organic farming system; CON, conventional farming system.

FIGURE S2 Beta-diversity of bacterial microbial communities in soils under organic (ORG) and conventional (CON) farming systems by UPGMA cluster analysis.

FIGURE S3 The linear relationship between the average dissimilarity of organic and conventional farming systems with the period of organic farming.

FIGURE S4 Beta-diversity of diazotrophic microbial communities in soils under organic (ORG) and conventional (CON) farming systems by UPGMA cluster analysis.

FIGURE S5 Beta-diversity of ammonia-oxidizing bacterial forward region microbial communities in soils under organic (ORG) and conventional (CON) farming systems by UPGMA cluster analysis.

FIGURE S6 Beta-diversity of ammonia-oxidizing bacterial reverse region microbial communities in soils under organic (ORG) and conventional (CON) farming systems by UPGMA cluster analysis.

FIGURE S7 Discriminative ammonia oxidizing reverse region OTUs between farming systems, which ranked in the 50 most influential OTUs. Brown and cyan squares indicate enrichment or depletion in the organic greenhouse farming system at the studied sites. ORG, organic farming system; CON, conventional farming system.

FIGURE S8 Dominant (>1% relative abundance) total, diazotrophic and ammonia-oxidizing bacterial OTUs and the fraction of genera by Co-occurrence network analysis.

FIGURE S9 Relative abundances of microbial hubs for total **(A)**, diazotrophs **(B)** and ammonia-oxidizing bacteria **(C)** by Co-occurrence network analysis.

FIGURE S10 The amount of variation in total, diazotrophic and ammonia-oxidizing bacterial communities explained by pH, soil types and farming systems.

TABLE S1 Details of agricultural managements under the long-term greenhouse experiments.
