# Supplementary figures and images for: Comparison of the Total, Diazotrophic and Ammonia-Oxidizing Bacterial Communities Between Under Organic and Conventional Greenhouse Farming
Source: Front Microbiol. 2020 Aug 12;11:1861. doi: 10.3389/fmicb.2020.01861 (PMC7434936; doi:10.3389/fmicb.2020.01861)

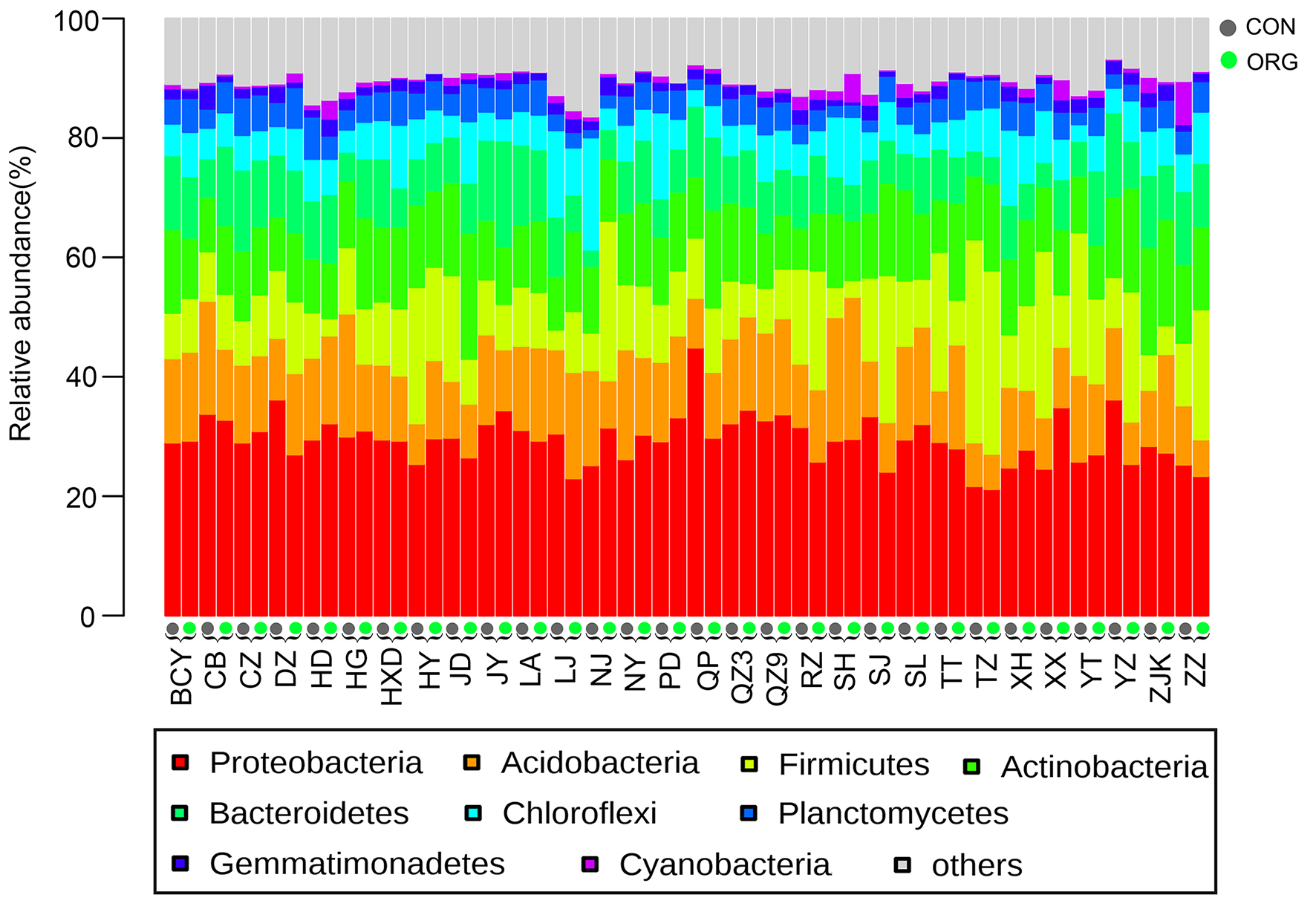

Supplement: FIGURE S1 — Relative abundances of dominant bacteria between farming systems. ORG, organic farming system; CON, conventional farming system. [file Image_1.TIF]

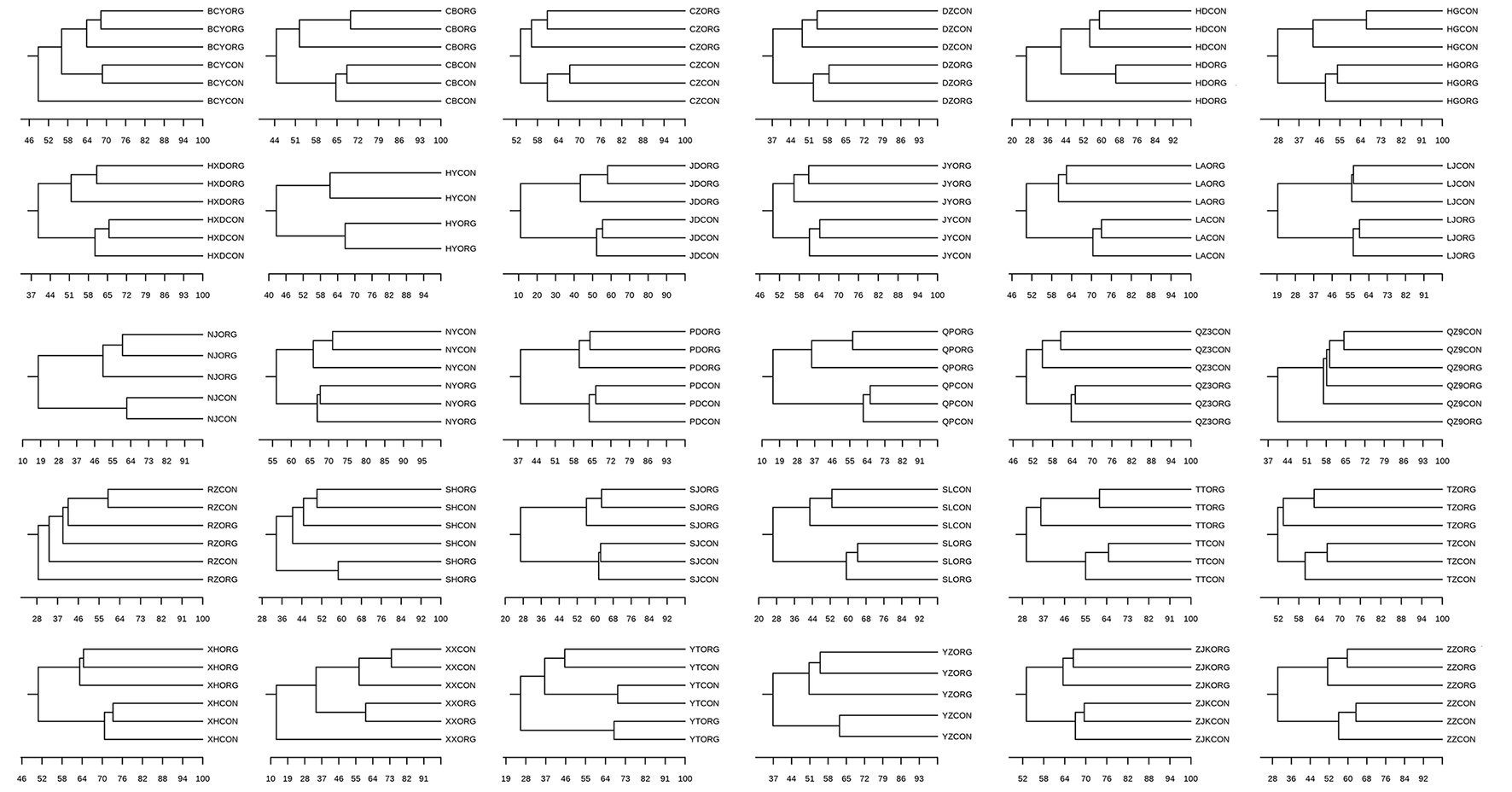

Supplement: FIGURE S2 — Beta-diversity of bacterial microbial communities in soils under organic (ORG) and conventional (CON) farming systems by UPGMA cluster analysis. [file Image_2.TIF]

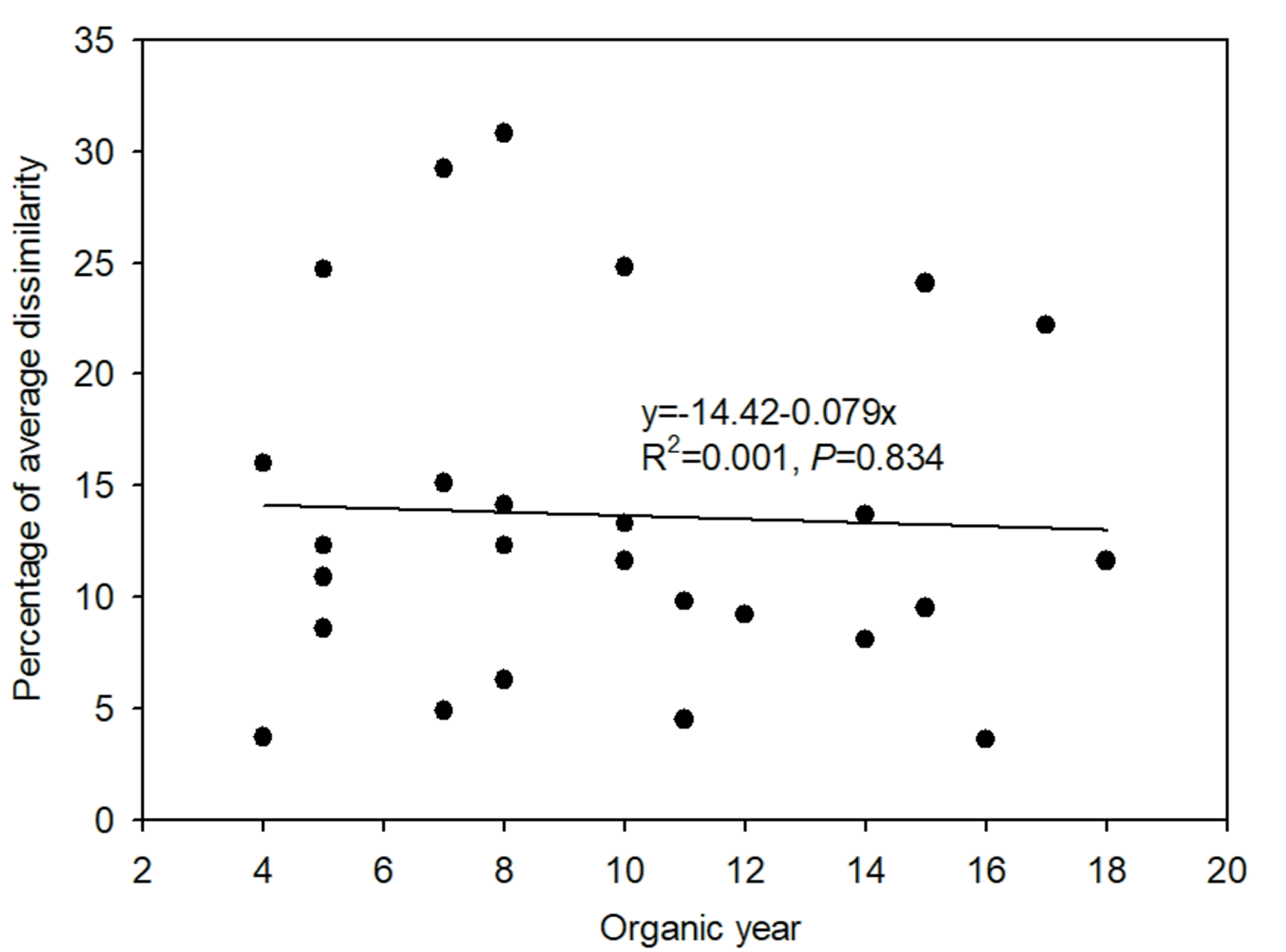

Supplement: FIGURE S3 — The linear relationship between the average dissimilarity of organic and conventional farming systems with the period of organic farming. [file Image_3.TIF]

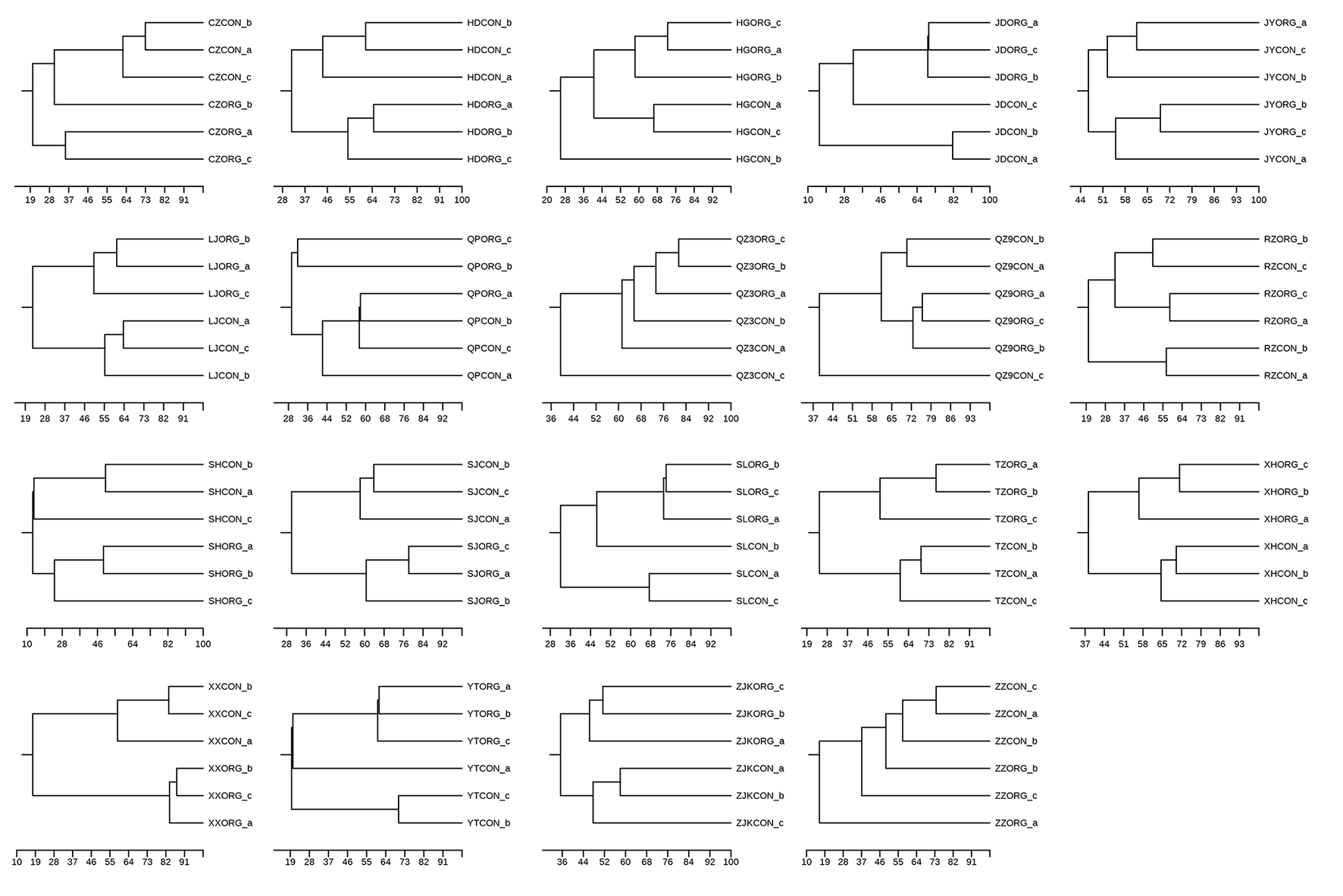

Supplement: FIGURE S4 — Beta-diversity of diazotrophic microbial communities in soils under organic (ORG) and conventional (CON) farming systems by UPGMA cluster analysis. [file Image_4.TIF]

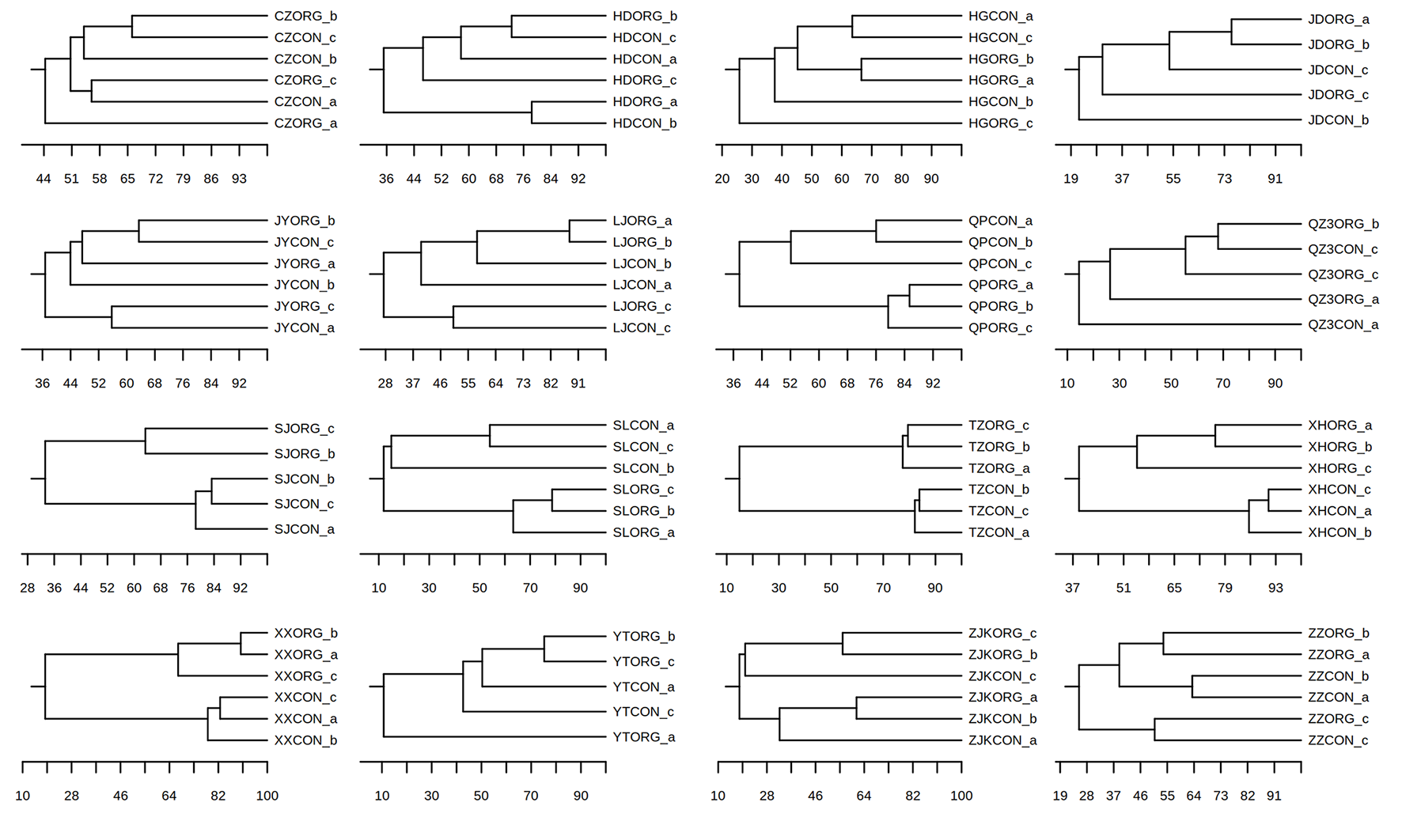

Supplement: FIGURE S5 — Beta-diversity of ammonia-oxidizing bacterial forward region microbial communities in soils under organic (ORG) and conventional (CON) farming systems by UPGMA cluster analysis. [file Image_5.TIF]

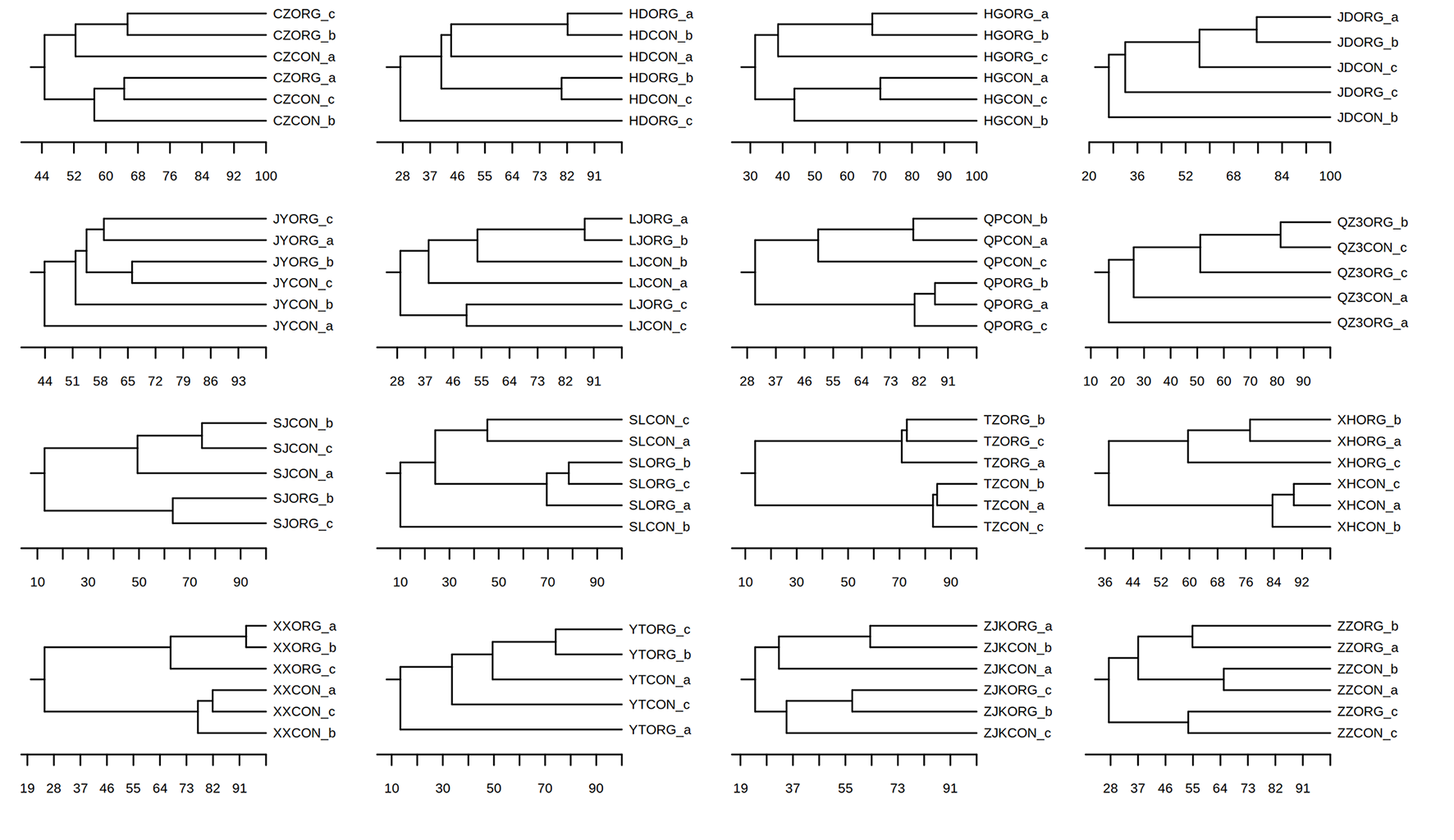

Supplement: FIGURE S6 — Beta-diversity of ammonia-oxidizing bacterial reverse region microbial communities in soils under organic (ORG) and conventional (CON) farming systems by UPGMA cluster analysis. [file Image_6.TIF]

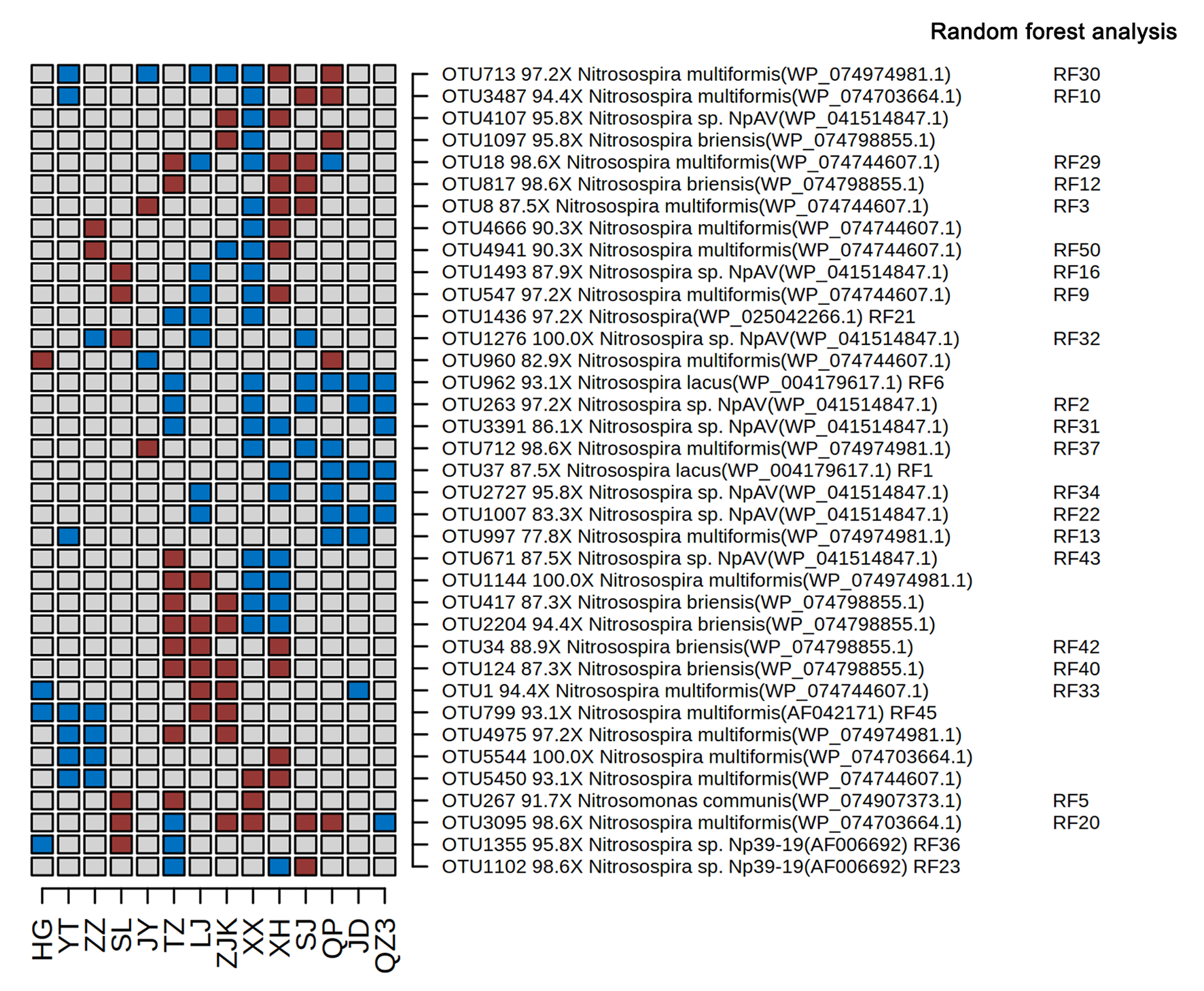

Supplement: FIGURE S7 — Discriminative ammonia oxidizing reverse region OTUs between farming systems, which ranked in the 50 most influential OTUs. Brown and cyan squares indicate enrichment or depletion in the organic greenhouse farming system at the studied sites. ORG, organic farming system; CON, conventional farming system. [file Image_7.TIFF]

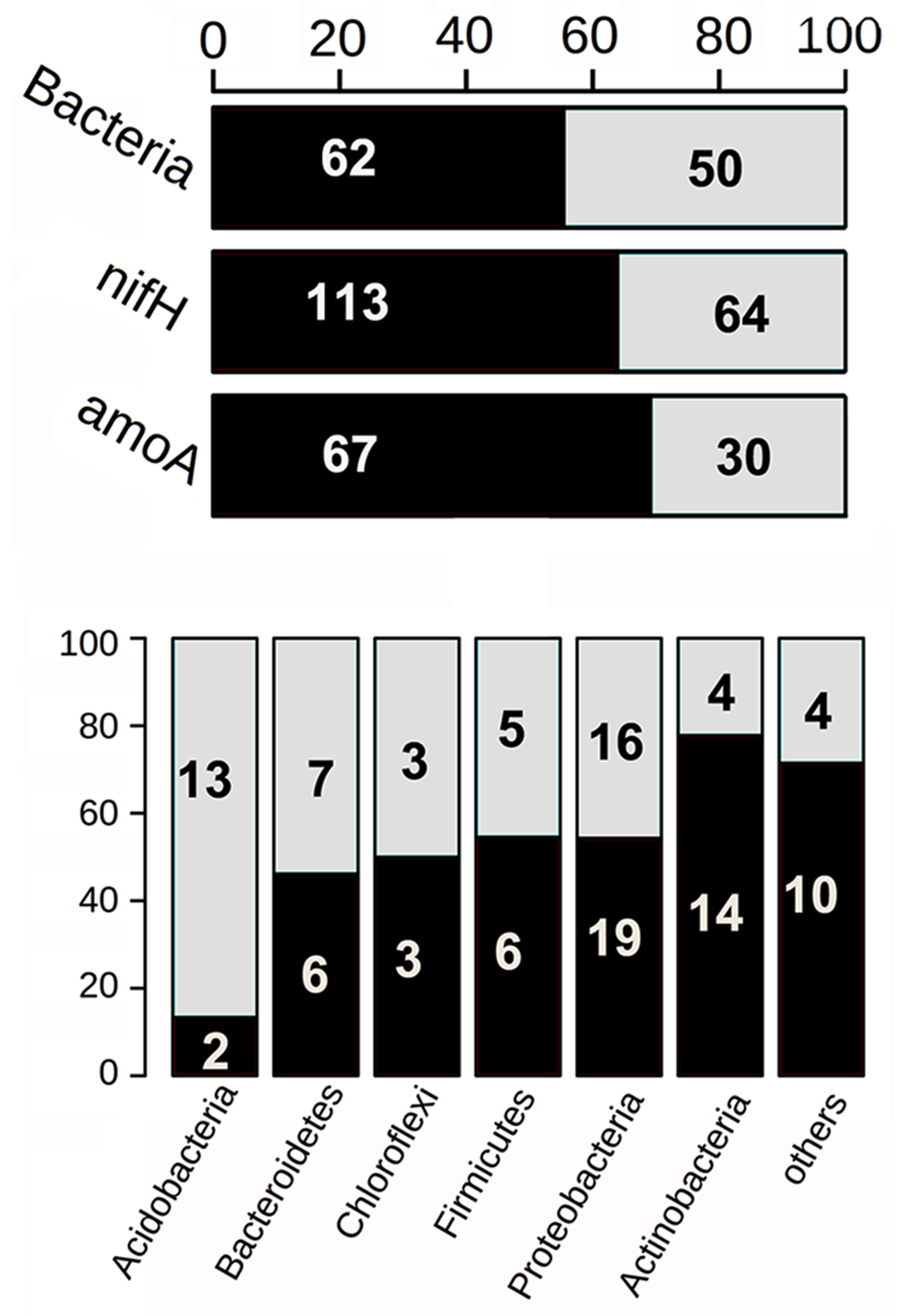

Supplement: FIGURE S8 — Dominant (>1% relative abundance) total, diazotrophic and ammonia-oxidizing bacterial OTUs and the fraction of genera by Co-occurrence network analysis. [file Image_8.TIF]

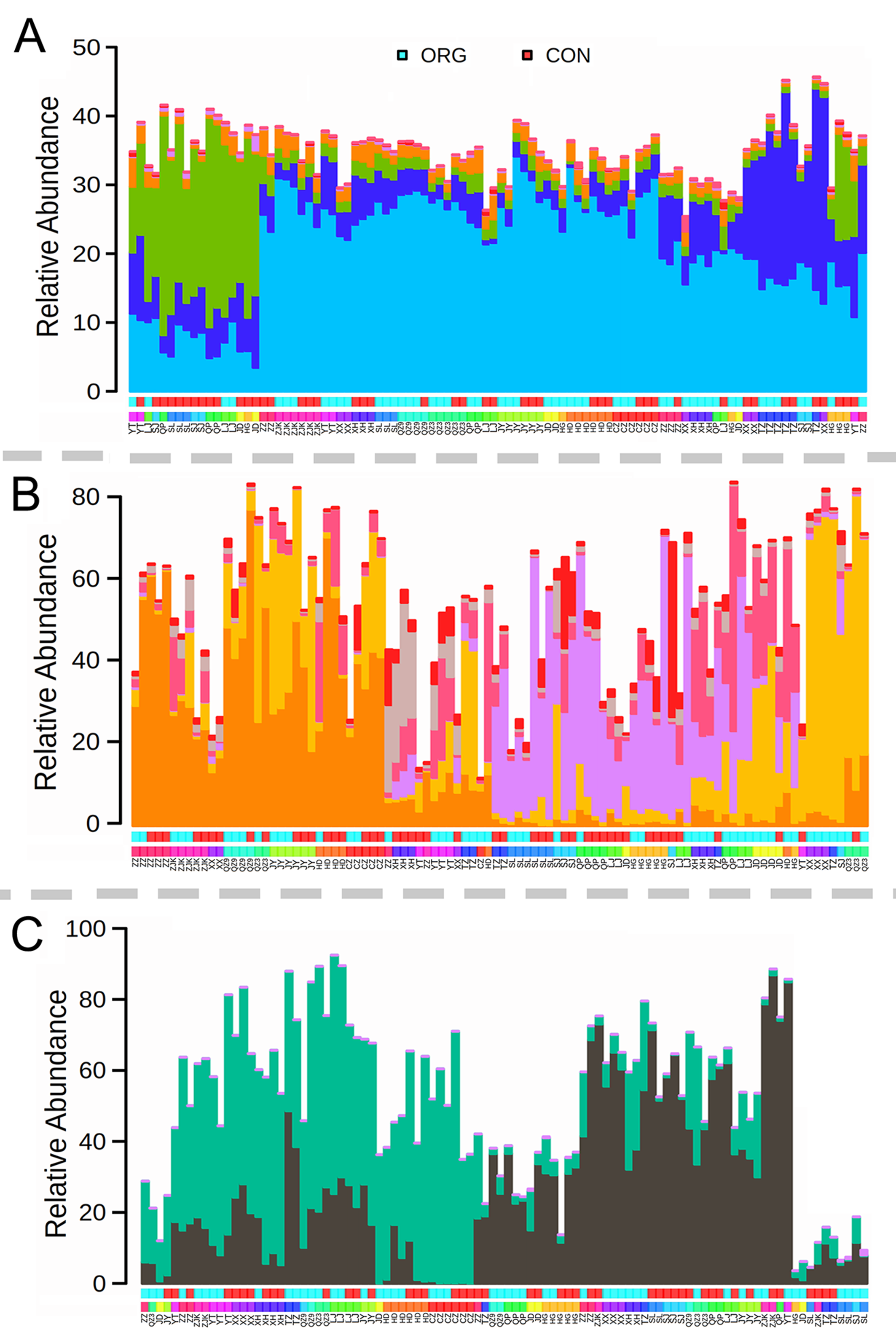

Supplement: FIGURE S9 — Relative abundances of microbial hubs for total (A), diazotrophs (B) and ammonia-oxidizing bacteria (C) by Co-occurrence network analysis. [file Image_9.TIF]

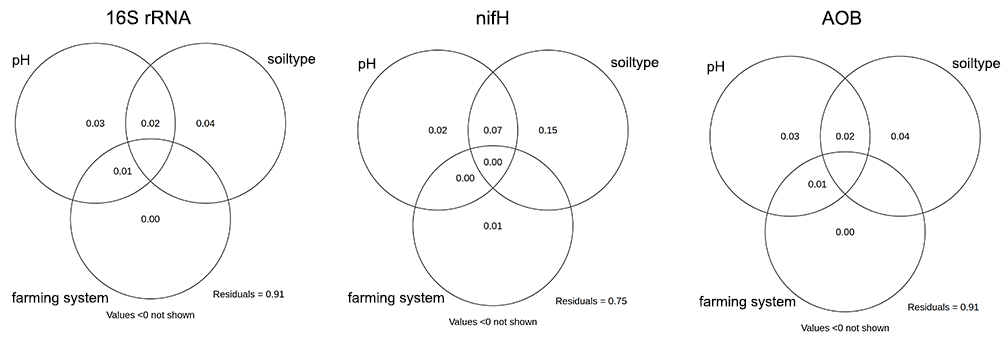

Supplement: FIGURE S10 — The amount of variation in total, diazotrophic and ammonia-oxidizing bacterial communities explained by pH, soil types and farming systems. [file Image_10.TIF]
