# Supplementary material for: Comparison of the Total, Diazotrophic and Ammonia-Oxidizing Bacterial Communities Between Under Organic and Conventional Greenhouse Farming
Source: Front Microbiol. 2020 Aug 12;11:1861. doi: 10.3389/fmicb.2020.01861 (PMC7434936; doi:10.3389/fmicb.2020.01861)
Supplement: TABLE S1 — Details of agricultural managements under the long-term greenhouse experiments. [file Table_1.docx]

Table S1 Primer sequences of functional genes in Illumina Hi-Seq high-throughput sequencing

| **Target genes** | **Primer name** | **Primer sequence** **(5'-3')** | **References** |
| --- | --- | --- | --- |
| *16S rRNA* | 515F | TGCGAYCCSAARGCBGACTC | Tamaki et al., 2011 |
|  | 909R | TGCGAYCCSAARGCBGACTC |  |
| *nifH* | PolF | TGCGAYCCSAARGCBGACTC | Poly et al., 2001 |
|  | PolR | ATSGCCATCATYTCRCCGGA |  |
| AOB*-amoA* | amoA-1F | GGGGTTTCTACTGGTGGT | Rotthauwe et al., 1997 |
|  | amoA-2R | CCCCTCKGSAAAGCCTTCTTC |  |
